# Micro-nano hierarchical urchin-like ZnO/Ag hollow sphere for SERS detection and photodegradation of antibiotics

**DOI:** 10.1515/nanoph-2023-0659

**Published:** 2024-01-23

**Authors:** Yang Jiao, Yuanyuan Pan, Moru Yang, Zhen Li, Jing Yu, Rong Fu, Baoyuan Man, Chao Zhang, Xiaofei Zhao

**Affiliations:** School of Physics and Electronics, Shandong Normal University, Jinan 250014, China; School of Chemistry and Chemical Engineering, Liaocheng University, Liaocheng 252000, China

**Keywords:** surface-enhanced Raman scattering, photodegradation, plasmonic, 3D ZnO/Ag hollow sphere, anti-biotics

## Abstract

Hollow urchin-like substrates have been widely interested in the field of surface-enhanced Raman scattering (SERS) and photocatalysis. However, most reported studies are simple nanoscale urchin-like substrate with limited light trapping range and complicated preparation process. In this paper, a simple and effective controllable synthesis strategy based on micro-nano hierarchical urchin-like ZnO/Ag hollow spheres was prepared. Compared with the 2D structure and solid spheres, the 3D urchin-like ZnO/Ag hollow sphere has higher laser utilization and more exposed specific surface area due to its special hollow structure, which resulted in excellent SERS and photocatalytic performance, and successfully realize the detection and photodegradation of antibiotics. The limited of detection of metronidazole can reach as low as 10^−9^ M, and degradation rate achieve 89 % within 120 min. The experimental and theoretical results confirm that the ZnO/Ag hollow spheres can be used in the development of ZnO heterostructure for the detection and degradation of antibiotics, which open new avenues for the development of novel ZnO-based substrate in SERS sensing and catalytic application to address environmental challenges.

## 1 Introduction

Surface-enhanced Raman scattering (SERS), as a label-free and highly sensitive analytical technique, has a wide range of applications in surface and interfacial chemistry [1], [2], biomedicine [3], food science and environmental analysis [4], [5], [6], [7], which has attracted more and more attention for researchers. It is well known that plasmonic nanoparticles (mainly Ag, Au, and Cu) can interact with light in the visible and NIR regions and strongly enhance electromagnetic fields, enable widespread applications in SERS sensing and photocatalysis [8], [9], [10], [11]. Due to their nanoscale size and nanogap, collective oscillation of electron clouds is limited on the surface of metal nanoparticles and nanostructured, resulting in unique optical properties known as localized surface plasmon resonance (LSPR) [12], [13]. Researchers have made many efforts to optimize the preparation methods and upgrade the structures of metal nanoparticles to improve substrate performance in recent decades [14], [15], [16], [17]. However, there are some considerable bottlenecks of plasmonic nanoparticle platforms, such as poor biocompatibility [18], low reproducibility, and inconvenient light loss [19], [20], which greatly limit the practical applications. Nevertheless, semiconductor materials have unique optical properties with good biocompatibility, stable chemical properties and low cost, which can effectively solve the above problem.

As a kind of metal oxide semiconductor, zinc oxide (ZnO) with a wide range of tunable morphologies possesses high electrons mobility and promotes strong light confinement, which has been recently exploited as a potential candidate for SERS sensor and photocatalysis. However, its wide bandgap (3.37 eV) and large exciton binding energy (60 meV) generate near UV emission and lead to low visible light absorption [21], [22], which considerably reduce the practical efficiency. Many efforts have been made by combining ZnO and metal nanoparticles to effectively improve the utilization of visible light. For example, Kang et al. [23] prepared two different asymmetric Ag/ZnO composite nanoarrays, where Ag nanoparticles (NPs) were hanging inside or capped on top of ZnO hollow nanospheres with high photocatalytic activity and SERS performance under visible light irradiation. Lin et al. [24] synthesize Au/ZnO SERS substrates for R6G detection by decorating Au NPs on the surface of ZnO nanorods using ion-sputtering, and the results showed that the composites exhibited good SERS sensitivity under visible light due to the multi-effect enhancement mechanism. However, just the material combination of ZnO and noble metal particles, the performance of heterostructure are restricted by their limited specific surface area and insufficient light utilization. Therefore, it is of great importance to develop effective optical substrates for SERS detection and photocatalysis with high sensitivity and selectivity.

It is generally known that the morphology of composite structures has a great influence on their optoelectronic properties [25], [26], [27]. Compared with one-dimensional (1D) structure, 3D architectures possess larger surface area, larger number of hot spots and stronger light-scattering ability [28], [29]. Among them, 3D urchin-like hollow sphere array represents an ideal photoelectrode candidate due to the excellent light trapping and direct charge transportation. Ye et al. [30] fabricated a sensitive SERS sensor of hollow sea urchin-like TiO_2_@Ag NPs functionalized with glutathione (GSH) and use 2-mercaptopyridine (2-MPy) as a Raman reporter, which enable realize ultrasensitive detection of Cr (VI). Yao et al. [31] developed a method for preparing hollow urchin-like Au crystals as bifunctional catalysts and SERS-enhancing materials to *in-situ* monitor the photocatalytic effect of 4-NTP with high photocatalytic efficiency and SERS performance. However, these structures reported above are simple nanoscale urchin-like hollow spheres with limited light trapping range and complicated preparation process, which restrict the actual development in photoelectric sensing field.

In this work, based on water bath growth and sacrificial template method, we successfully prepared a 3D micro-nano hierarchical urchin-like ZnO hollow sphere array composited with Ag NPs (ZnO/Ag HS) as a novel bifunctional substrate for SERS sensing and photocatalytic degradation. Herein, the micro-HS structure composed by ZnO nanorods provides richer optical coupling forms, which can improve the light capture ability and increase enrichment efficiency of wide-band light. In addition, the plasmonic Ag NPs offer strong electromagnetic field and enhance visible-light absorption. We demonstrated the ZnO/Ag HS heterostructure have higher SERS intensity for antibiotics detection and better photocatalytic efficiency for antibiotics degradation than solid sphere (SS) due to excellent light capture capability and more exposed active sites. Furthermore, the uniformity and reproducibility of the ZnO/Ag HS were proved by multiple positions and multiple batch detection. The experimental and theoretical results indicated that the proposed 3D urchin-like ZnO/Ag HS structure shows great advantages in improving visible light absorption performance, which can be used as a promising material for low concentration molecule detection and antibiotics photocatalytic degradation.

## 2 Experimental section

### 2.1 Materials

In all experiments, deionized (DI) water was ultra-pure and came from the PSDK2-20-D water purification system. Silicon (Si) wafers of 10 × 10 mm^2^ were obtained from Zhejiang Lijing. PS spheres (5 μm) were purchased from Rigel Technologies. Ethanol was purchased from Dexcom, zinc acetate was purchased from Solaibio. Methyl green (MG), crystal violet (CV), and ciprofloxacin (CIP) was obtained from Macklin. Hexamethylenetetramine, rhodamine 6G (R6G), and metronidazole (MNZ) was purchased from Aladdin.

### 2.2 Preparation of 3D urchin-like ZnO/Ag HS

Firstly, 10 µL ethanol mixture of PS spheres (volume ratio 1:1) was dropped onto a 10 × 10 mm^2^ Si wafer treated with a plasma cleaner, following spin by spin coater with speed of 3000 r/min for 30 s, and then placed on a heating platform at 110 °C for 1 min to make the PS spheres firmly attached to the wafer. Whereafter, the Si wafer with neatly arranged PS spheres was dipped into 5 mM Zn(C_2_H_3_O_2_)_2_ ethanol solution for 10 s and place on a heating platform at 90 °C for 40 min, which was repeated 3 times to form ZnO seed on the surface of PS microspheres. The Si wafers were then placed in a beaker containing a mixture of Zn(C_2_H_3_O_2_)_2_ and hexamethyl tetramine solution, water bath heating at 95 °C for 1 h, following washed with DI water and dried naturally to obtain ZnO SS. The ZnO HS was prepared by thermal annealing at 400 °C for 1 h to remove PS microspheres. Finally, Ag was deposited on obtained ZnO HS by thermal-evaporation method with vaporization rate of 0.2 Å/s at 5 × 10^−4^ Pa, forming 3D urchin-like ZnO/Ag HS.

### 2.3 SERS detection

A total of 2 µL of probe molecules aqueous solution (R6G, MNZ, CV, and MG) with different concentration was dropped on the surface of sample and dried in air. To avoid detection errors, we detected SERS signal from random 10 position and calculate the average intensity. The Raman signals of the probe molecules were recorded using Horiba HR Evolution 800 Raman spectrometer with a laser wavelength of 532 nm/633 nm (R6G: 532 nm with laser power of 0.048 mW, MNZ: 532 nm with laser power of 0.48 mW, CV and MG: 633 nm with laser power of 0.048 mW). The accumulation time was 4 s and diffraction grid was 600 g/mm.

### 2.4 Photocatalytic applications research

20 × 20 mm^2^ substrates were, respectively, immersed in 30 mL metronidazole, ciprofloxacin, and norfloxacin aqueous solution at an initial concentration of 20 mg/L and irradiated with a xenon (Xe) lamp of 294 W power for 2 h. Before the photocatalytic degradation, the substrate was placed into 20 mg/L metronidazole/norfloxacin/ciprofloxacin aqueous solution for 30 min under dark environment to reach adsorption–desorption equilibrium. During light irradiation, the solution of 30 mL was extracted from the reactor every 20 min. The catalytic activity was analyzed by UV–vis spectrophotometer and determined by recording the UV–vis spectra of antibiotic solution at different irradiation times. The height and position of the sample with respect to the incident light source are stationary to ensure consistent light intensity for all antibiotic degradation experiments.

### 2.5 Characteristic description

Scanning electron microscopy (SEM) images were taken using a Sigma 500 thermal field emission electron microscope with a primary electron energy of 3 kV, which is equipped with an energy dispersive spectrometer (EDS) for elemental analysis. Before SEM detection, a thin Au film (approximate thickness of 2 nm) was sputtered to enhance the electrical conductivity of the samples. UV–vis absorption spectra were collected by a UV–vis spectrophotometer (SHIMADZU, RealSpec-3700i DUV). Meanwhile, we characterized the phase structure of the surface using X-ray diffraction (XRD, D8 Advance). X-ray photoelectron spectroscopy (XPS, ESCALAB Xi+) was used to analyze the chemical state of the surface of prepared substrate.

## 3 Results and discussions

### 3.1 Characterization of 3D urchin-like ZnO/Ag HS structure

**Figure 1:**
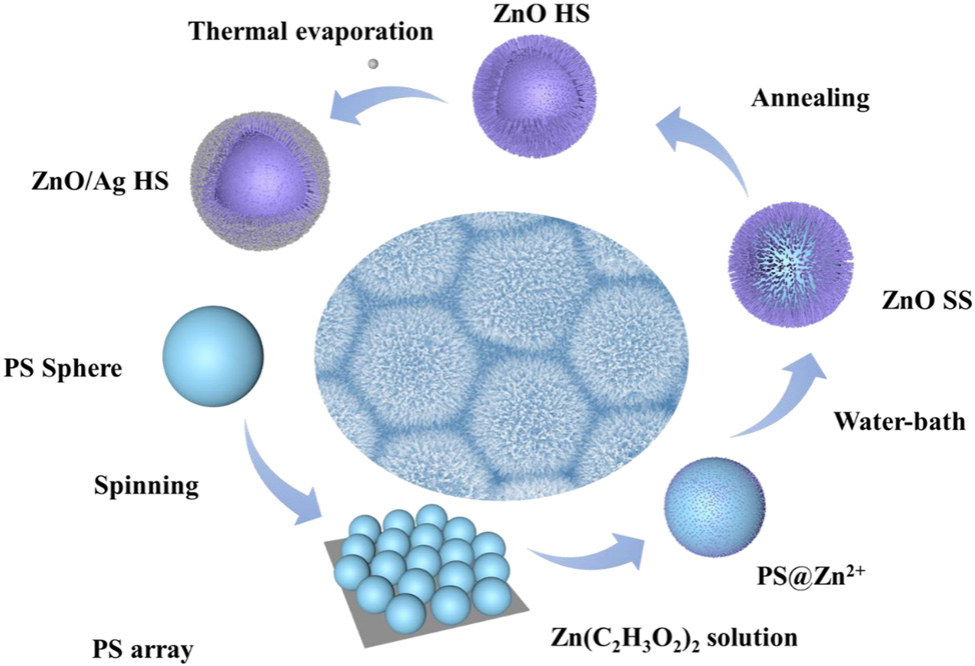
Schematic of the fabrication process.

The synthetic process of 3D urchin-like ZnO/Ag HS was schematically illustrated in Figure 1. Briefly, the PS microspheres with diameter of 5 μm were firstly self-assembled on the plasma-treated Si substrate by the spin-coating method, and then the prepared substrate was heated at 110 °C for 1 min to stabilize PS microspheres on Si. After stabilization, the ZnO seed solution was spread on the surface of PS by repeatedly dip-coating of Zn(C_2_H_3_O_2_)_2_ solution, and then the ZnO nanorods were uniformly grown on ordered PS microsphere templates by the water-bath method. Whereafter the PS spheres were removed by the thermal annealing, forming ZnO HS. Finally, 3D urchin-like ZnO/Ag HS structure was synthesized after Ag deposition. A photograph of the prepared real substrate was shown in Figure S1.

**Figure 2:**
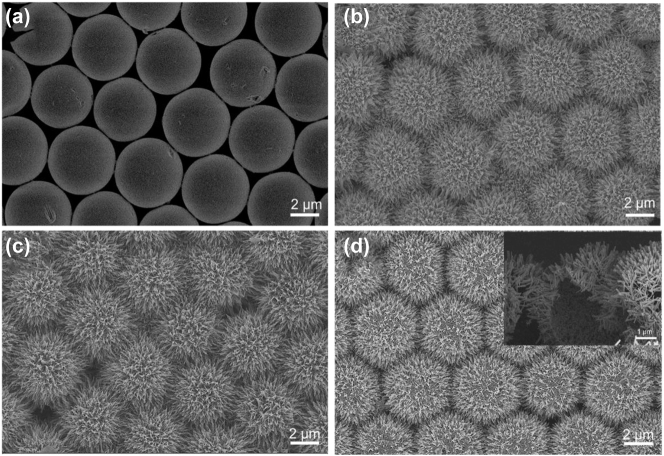
Top-view SEM images of (a) tiled PS spheres and (b) 3D urchin-like ZnO SS structure. (c) Top-view SEM images of 3D urchin-like ZnO HS structure. (d) 3D urchin-like ZnO/Ag HS structure, and the inset of (d) was cross-sectional SEM images of 3D urchin-like ZnO/Ag HS structure.

The scanning electron microscope (SEM) images with different substrate are shown in Figure 2 to characterize micromorphology. Figure 2a shows the compactly arranged PS microspheres obtained by the spin-coating method, where all the microspheres were uniformly assembled on Si wafer with diameter of 5 µm. After growing ZnO nanorods on PS by water-bath method, the morphology of urchin-like ZnO SS was shown in Figure 2b, it can be clearly observed the ZnO nanorods vertically aligned and uniformly grown around the sphere forming urchin-like structure. Figure 2c presents the microstructure of the 3D ZnO HS structure after annealing to remove the PS microspheres, and it has no much difference with ZnO SS in the top-view SEM images except the ZnO nanorods become sharper, which is conducive to the generation of more hot spots. The results indicated the process of annealing has no effect on the spherical structure Figure 2d show the SEM of the 3D ZnO/Ag HS structure from top view, where the morphology of urchin-like structure has no significant changes after deposition of Ag NPs. To verify the hollow structure, we cut off the substrate from optional position. The cross-sectional view was illustrated in the inset of Figure 2d, we can clearly see that the PS spheres have been completely removed and only the self-supporting ZnO/Ag nanorod hollow sphere with diameter of 5 µm (consistent with PS sphere template) was remained, which means that we have successfully prepared the 3D urchin-like HS structure array. Figure S2 shows the corresponding EDS elemental mapping, and we can see that the elements of Ag, Zn, and O are evenly distributed on the surface of ZnO/Ag HS.

**Figure 3:**
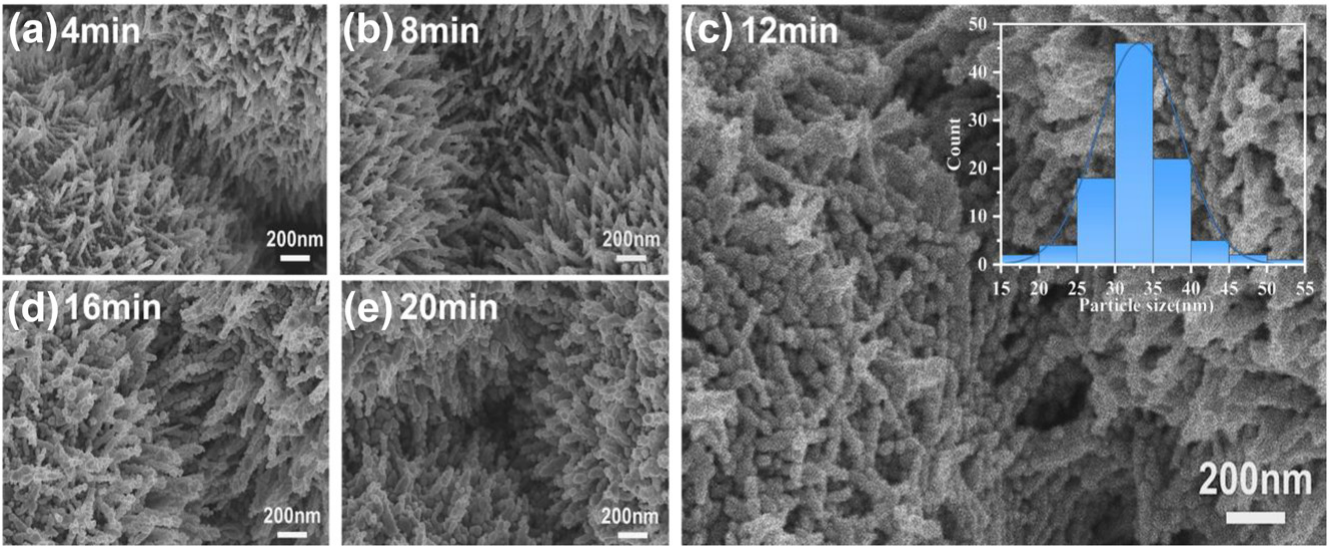
SEM images of Ag NPs with deposition time of (a) 4 min, (b) 8 min, (c) 12 min, (d) 16 min, and (e) 20 min on the surface of ZnO nanorods respectively. The inset of (c) is normal distribution of the diameter of Ag NPs with 12 min deposition time.

Figure 3 shows typical SEM images of 3D urchin-like ZnO/Ag HS substrate modified with deposition of Ag for different deposition times of 4 min, 8 min, 12 min, 16 min, and 20 min respectively. With increasing deposition time of Ag, the top and sidewalls of the ZnO nanorods were covered by Ag NPs more and more sufficiently. Due to the Volmer–Weber (VW) growth mode, the Ag deposited at short time formed small Ag NPs on the sidewalls of the ZnO nanorods with large spacing between neighboring Ag NPs [32], [33]. With increasing Ag deposition time on the surface, the size of Ag NPs enlarges and the nano-gap spacing decrease. By further increasing Ag deposition time, the coalescence between Ag NPs come up and resulted in the formation of aggregate structure. The SEM in Figure 3a–e clearly show the gradual change of Ag NPs located on the sidewalls of ZnO nanorods from 4 min to 20 min. The Ag NPs is denser with deposition time increase before 12 min, whereafter the aggregation happened at 16 min and the ZnO was completely covered by Ag at 20 min. The inset in Figure 3c shows the normal distribution of the diameter of Ag NPs at deposition time of 12 min with an average size of about 33.22 nm, which is the main plasmonic hot spot that promotes Raman enhancement.

**Figure 4:**
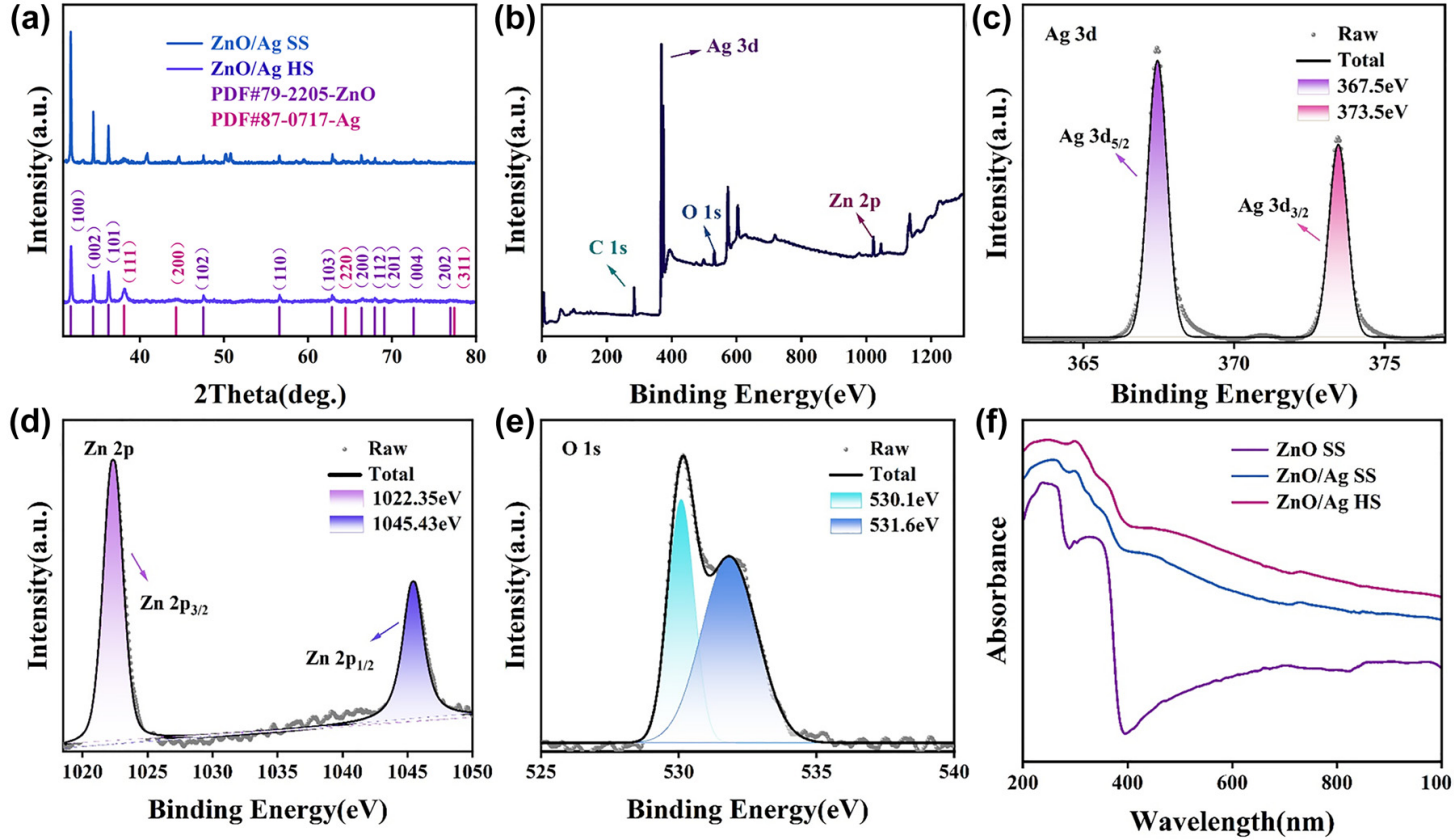
Element characterization of substrates. (a) XRD comparison image of 3D urchin ZnO/Ag HS structure and ZnO/Ag SS structure. (b–e) XPS of 3D urchin ZnO/Ag HS structure: (b) full spectrum, (c) Ag 3d, (d) Zn 2p and (e) O 1s. (f) UV-visible absorption spectra of different substrates.

To study the specific structure and chemical morphology of the substrate, XRD and XPS were carried out in Figure 4. The XRD spectra of the substrate before and after annealing are presented in Figure 4a. The strong and sharp peaks at 2*θ* values of 31.7, 34.4, 36.2, 47.5, 56.6, 62.9, 66.4, 67.9, 69.1, 72.6, and 77, which are corresponding to (100), (002), (101), (102), (110), (103), (200), (112), (201), (004), and (202) crystal planes of the hexagonal ZnO structure, respectively [34]. All these peaks of ZnO photocatalysts can be indexed to the wurtzite structure of ZnO (PDF#79–2205). Besides, in the case of Ag NPs decorated ZnO photocatalysts additional peaks at 38.1, 44.3, 64.5, and 77.4 are visible, which are assigned to (111), (200), (220), and (311) crystal planes of Ag NPs, respectively [35]. This finding supported the decoration of Ag NPs on ZnO photocatalysts. Moreover, we can see that only the diffraction peak of ZnO material (PDF#79–2205) and the diffraction peak of metal Ag (PDF#87–0717) existed on the ZnO/Ag HS, indicating that PS spheres have been removed completely without any impurity. The total XPS spectrum of the ZnO/Ag HS (annealed substrate) in Figure 4b shown the characteristic peaks of C 1s, Ag 3d, O 1s, and Zn 2p were apparent. Among them, the appearance of the C 1s peak at 284.8 eV was due to the test instrument itself introducing foreign C sources to standardize the position of other elements. Figure 4c–e shows the high-resolution spectra of Ag 3d, Zn 2P, and O 1s from ZnO/Ag substrate respectively. From XPS spectrum of Ag 3d in Figure 4c, we can see the presence of two separate peaks at 367.5 eV and 373.5 eV corresponding to the Ag 3d_5/2_ orbital and the Ag 2d_3/2_ orbital, respectively. The spacing between the two peaks is 6 eV, indicating that Ag is present in the form of Ag monomers [36], [37]. The Zn 2p related peaks (Figure 4d) at the horizontal coordinates located at 1022.35 eV and 1045.35 eV belong to the XPS characteristic peaks of the Zn 2p_3/2_ and Zn 2p_1/2_ orbitals, respectively, where the energy distance between Zn 2p_3/2_ and Zn 2p_1/2_ is 23 eV consistent with from of ZnO [36], [37]. In Figure 4e, two peaks appeared at binding energies of 530.1 eV and 531.6 eV, corresponding to the crystal lattice oxygen in ZnO and surface hydroxyl groups (vacancy oxygen) [38], [39], which facilitates the capture of photo-induced electrons and holes, thus enhancing the SERS sensitivity and photocatalytic degradation efficiency [40]. To illustrate the excellent optical absorption capability of the substrates, the UV-visible absorption spectra of different substrates were compared in Figure 4f, which shows that the ZnO SS substrates have a poor absorption intensity in the visible light region. After Ag deposition, the ZnO/Ag SS have a significant enhancement from 400 nm to 1000 nm. Particularly, the obvious absorption peak at 475 nm arises from the LSPR property of Ag NPs [41]. In addition, the 3D urchin-like ZnO/Ag HS structure has a better light absorption capacity than the ZnO/Ag SS structure benefited from the fact that a large number of photons can enter the inner cavity of the substrate and be reflected several times [42], [43], [44], which is beneficial to the capture and efficient utilization of light.

### 3.2 SERS properties of 3D urchin-like ZnO/Ag HS structure

**Figure 5:**
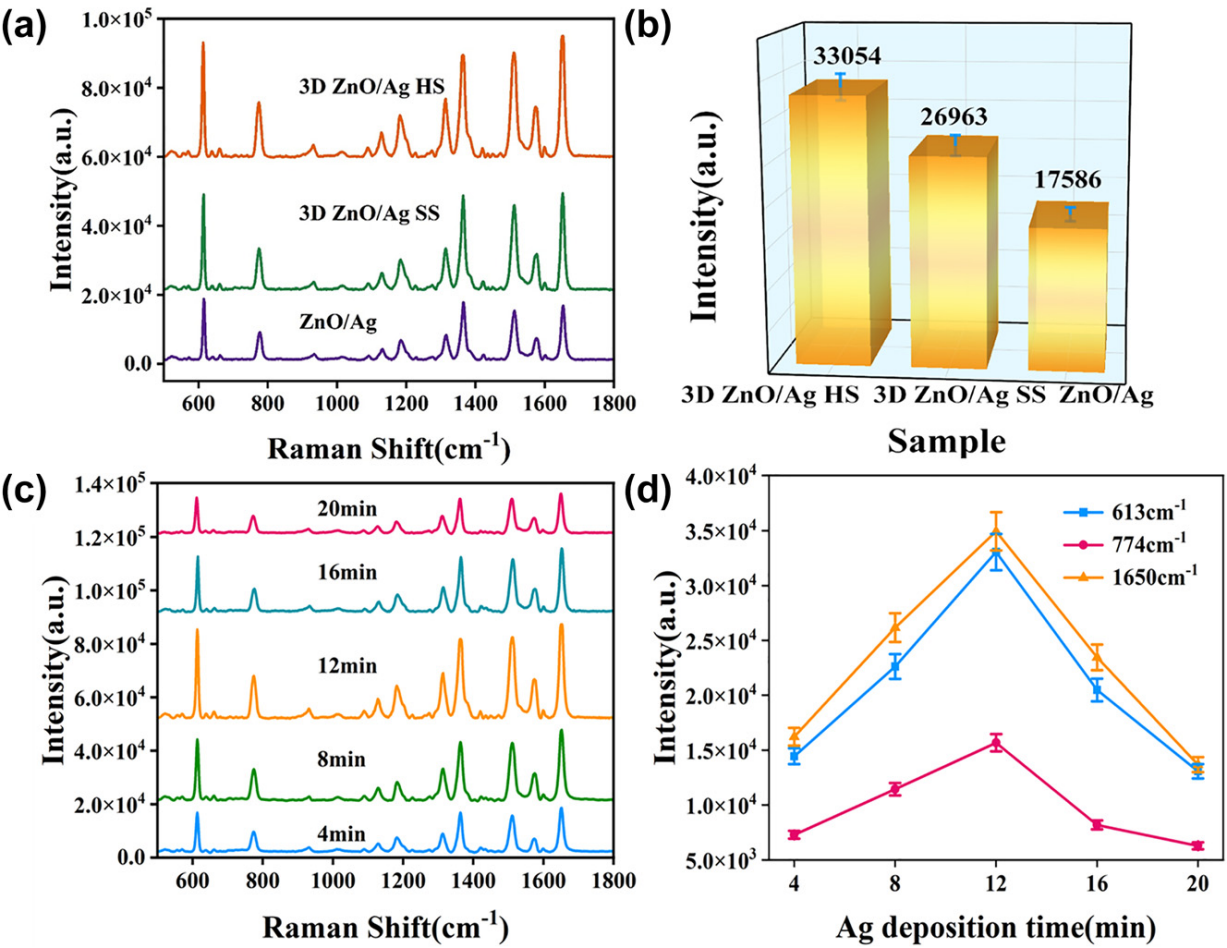
Raman characterization images of different substrates. (a) Raman spectra of R6G at a concentration of 10^−5^ M on different ZnO array substrates. (b) The histogram of Raman intensity at 613 cm^−1^ for different ZnO array structures. (c) Raman spectra of 10^−5^ M R6G on 3D urchin-like ZnO/Ag HS substrates with different Ag deposition time. (d) The Raman intensity of peaks at 613, 774, and 1650 cm^−1^ corresponding to different Ag deposition time.

To evaluate the micro-nano hierarchical urchin-like structural advantage in SERS detection, conventional 2D ZnO nanorod arrays and 3D urchin-like ZnO/Ag SS structure were compared with 3D urchin-like ZnO/Ag HS, and 10^−5^ M R6G molecules were used as probe molecules. As shown in Figure 5a, the 3D urchin-like ZnO/Ag HS structure has the highest SERS intensity compared with the 2D ZnO nanorod array and the SS structure, indicating the micro-nano hierarchical hollow structure possess more hot spots and better SERS performance. Figure 5b shows the Raman intensity with error bar of 10^−5^ M R6G probe molecule at 613 cm^−1^, which more visually demonstrated the excellent SERS performance of the 3D urchin-like ZnO/Ag HS structure. Compared with the 2D array structure, the 3D SS structure possesses bigger specific surface area and better SERS enhancement. While the 3D HS with micro-nano hierarchical structure obtains stronger light absorption through reflection and scattering from the inner cavity to improve the light utilization, thus have the best detection sensitivity.

To explore the optimal SERS enhancement conditions, the ZnO/Ag HS substrate with different deposition time of Ag was used for 10^−5^ M R6G detection. As shown in Figure 5c, the obvious characteristic peaks of 613, 774, 1365, 1508, and 1650 cm^−1^ can be observed on ZnO/Ag HS substrate, which are in varying degrees of intensity. To display the changing trend more clearly, Figure 5d describes the line graphs of the corresponding main Raman peak intensities at 613, 774, and 1650 cm^−1^, which depend on the Ag deposition time. The relative intensities at 613, 774, and 1650 cm^−1^ all increased at first and then decreased with Ag deposition time from 4 min to 20 min, and had the best enhancement at 12 min profited by the optimal particle size and space. The results illustrated the 3D urchin-like ZnO/Ag HS substrate with Ag deposition time of 12 min exhibits the highest SERS performance, which is fixed experimental condition for the following measurement.

**Figure 6:**
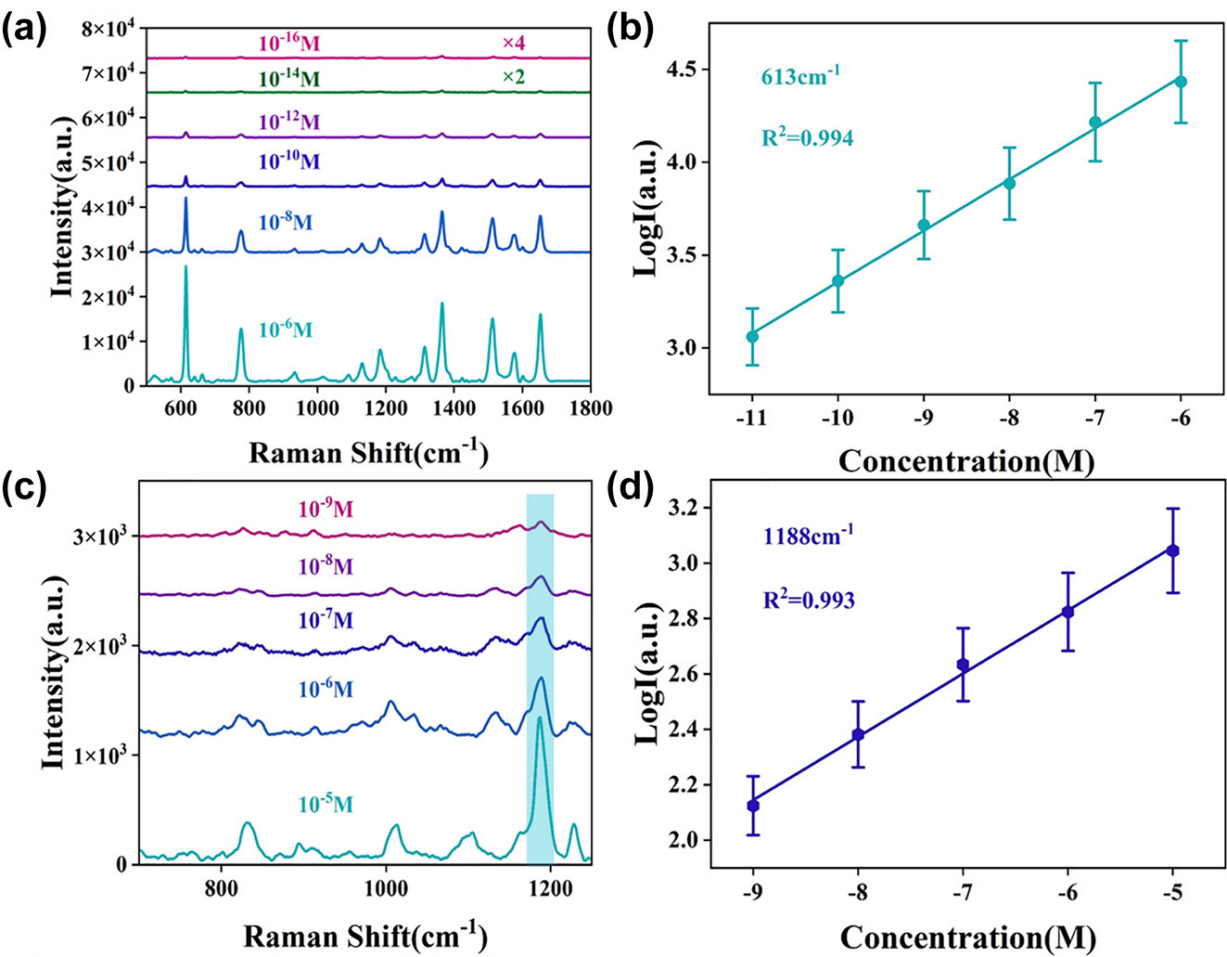
Raman spectra on 3D urchin-like ZnO/Ag HS structure. (a) Raman spectra of different concentrations of R6G on 3D urchin-like ZnO/Ag HS structure. (b) The calibration curves of Raman intensity versus R6G concentration (10^−6^–10^−11^ M) at 613 cm^−1^. (c) Raman spectra of different concentrations of MNZ on 3D urchin-like ZnO/Ag HS structure. (d) The calibration curves of Raman intensity versus MNZ concentration (10^−5^–10^−9^ M) at 1188 cm^−1^.

To further investigate the sensitivity of ZnO/Ag HS structure, Figure 6a shows the Raman spectra of R6G molecules with different concentrations on the SERS substrate under the same conditions, which all the Raman spectra of R6G from 10^−6^ to 10^−16^ M have characteristic peaks. The Raman intensity weakens as the concentration of R6G decreases, while at super low concentration of 10^−16^ M, the main characteristic peaks at 613 cm^−1^ and 774 cm^−1^ can still be observed, further demonstrating the high sensitivity of the 3D urchin-like ZnO/Ag HS structure. The limited of detection (LOD) of ZnO/Ag HS is much lower than other works about ZnO composited Ag NPs (down to 10^−12^ M–10^−13^ M) [45], [46], indicating the hollow sphere structure has unique advantages in SERS detection. Furthermore, the linear fit correction curve at 613 cm^−1^ with error bars was shown in Figure 6b, where the intensity of the SERS spectrum from 10^−6^ to 10^−11^ M is positively proportional to the R6G concentration under log scale with correlation coefficient (*R* ^2^) of 0.994. Except for R6G, the CV and MG molecule also be detected on urchin-like ZnO/Ag HS, the Raman spectra were shown in Figure S3a and b respectively. Even the concentration of probe molecule was as low as 10^−11^ M, the main characteristic peak of CV at 913, 1175, and 1620 cm^−1^ and MG at 1174, 1394, and 1616 cm^−1^ can still be observed. Figure S3c and d show the excellent linear fit correction curves of CV at 913 cm^−1^ (*R* ^2^ = 0.995) and MG at 1616 cm^−1^ (*R* ^2^ = 0.993) with error bars, respectively. The experiment results show that the ZnO/Ag HS have a great potential for qualitative and quantitative dye molecule detection.

Gradually, antibiotics have become the focus of human beings and are widely used in medical, animal husbandry and agricultural production because of their antibacterial properties and low side effects [47], [48]. Metronidazole (MNZ) is one of the most effective antibiotics for humans and animals, but overuse can also lead to an increased risk of cancer or genetic mutations [49]. Therefore, it is very important to detect MNZ qualitatively and quantitatively. Given the excellent SERS performance of 3D urchin-like ZnO/Ag HS structure, the SERS substrate was performed to detect MNZ molecule, and Figure 6c shows the Raman spectra of MNZ molecules from 10^−5^ to 10^−9^ M, where the characteristic peaks at 1188 cm^−1^ were observed even as low as 10^−9^ M. The linear fit correction curve at 1188 cm^−1^ with error bars (*R* ^2^ = 0.993) was shown in Figure 6d, showing the HS heterostructure can realize quantitative detection for MNZ molecule. These results manifested micro-nano hierarchical urchin-like ZnO/Ag HS was a perfect substrate for antibiotics detection.

Besides sensitivity, the uniformity and reproducibility of the SERS substrate play an important role in practical applications. As shown in Figure S4a, we measure 400 Raman spectra of 10^−5^ M R6G on ZnO/Ag HS with step size of 2 μm, where the nearly consistent color illustrate the Raman intensity have only slight fluctuation with relative standard deviation (RSD) of 2.93 %, indicating the ZnO/Ag HS substrate possess excellent uniformity. In addition, Figure S4b shows the relative relationship of intensities at 613 cm^−1^ from 10 batches ZnO/Ag HS. All relative intensities of R6G exhibit slight variations around the average value with RSD of 3.92 %. Long-term stability was assessed by randomly detecting 20 points (average intensity) of 10^−5^ M R6G using the same SERS substrate at day 1, day 14, and day 28. As shown in Figure S4c, the Raman signal of the substrate showed no significant decrease when the substrate was stored in a vacuum environment for 14 days, and the signal at 613 cm^−1^ decayed by 22 % only on day 28 (as shown in Figure S4d). These results indicate that uniform Raman signal and good reproducibility can be achieved on 3D urchin-like ZnO/Ag HS substrate.

**Figure 7:**
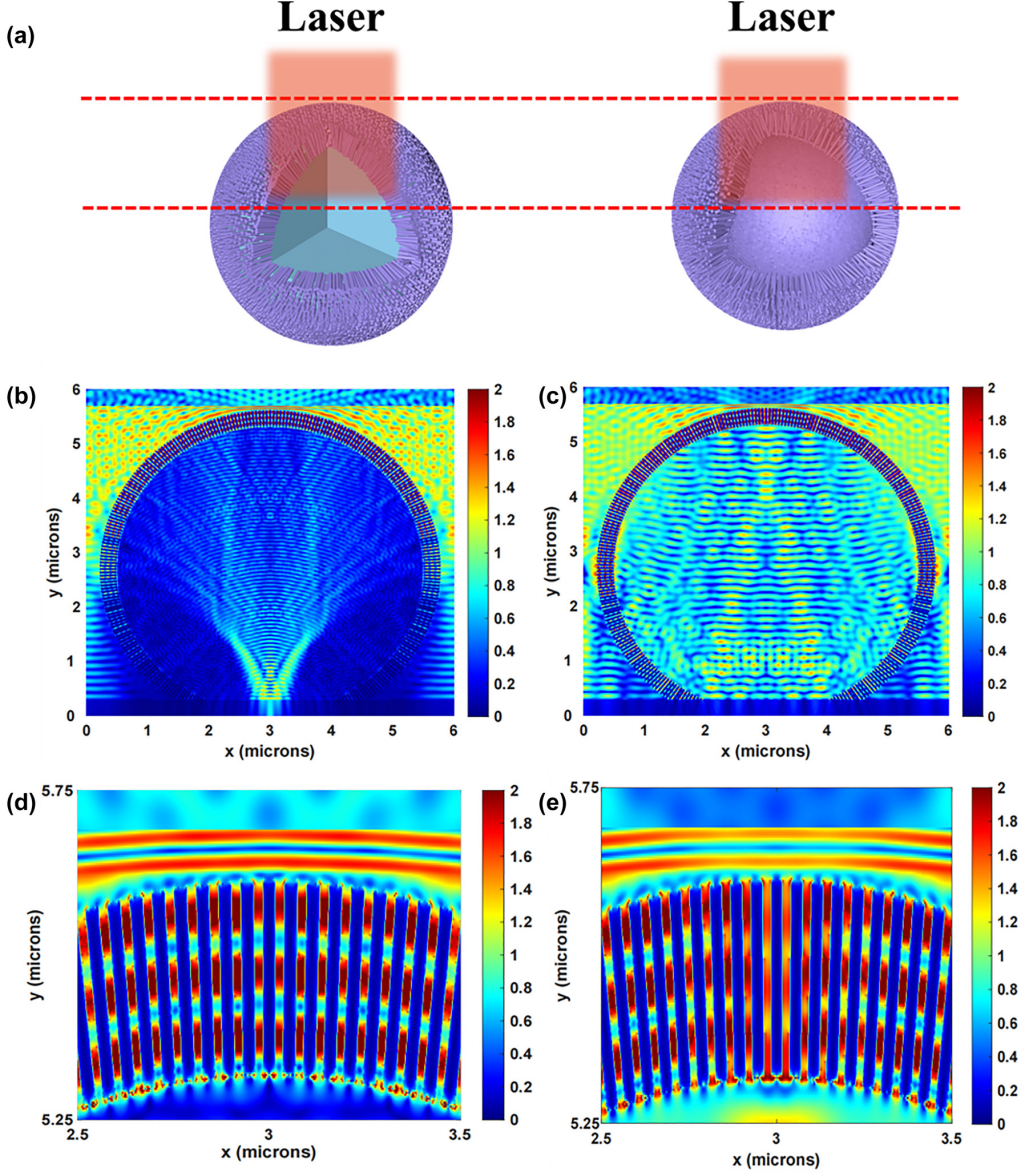
FDTD simulation of 3D ZnO SS structure and HS structure. (a) The schematic comparison between 3D ZnO SS structure and HS structure. Calculated the EM field distribution of (b) 3D urchin-like ZnO SS structure and (c) 3D urchin-like ZnO HS structure by FDTD method under 532 nm laser illumination. (d) and (e) Correspond to the magnifying EM field distribution from (b) and (c), respectively.

To demonstrate the superiority of hollow sphere structure, the finite-difference time-domain (FDTD) simulations were used to understand the SERS enhancement mechanism. The normalized electric field intensity distributions of the 3D urchin-like ZnO SS and HS structures under 532 nm laser illumination are shown in Figure 7. Figure 7a illustrated the schematic comparison between ZnO SS and ZnO HS. The utilization of incident light on ZnO HS is improved due to the 3D microscopic environment provided by the microsphere array below. In addition, the ZnO nanorods array maintains a certain degree of mutual independence, which greatly reduces the consequences of assembly defects. Figure 7b and c shows the electric field simulations for the 3D urchin-like ZnO SS and HS structures respectively. From the calculation results, the HS structure shows a large number of hot spots in the inner cavity compared to the SS structure profited by the light limit effect of hollow structure. In addition, it can be seen the enlarged electric field distribution in Figure 7d and e, where the electric field intensity between the ZnO nanorods on the HS structure is also higher than that on the SS structure, which was attributed to the inner hollow sphere for multiple light reflections and scattering, and the coupling effect between the micro cavity and nano ZnO.

### 3.3 Catalytic properties of 3D urchin-like ZnO/Ag HS structure

As we all known, more than 75 % of antibiotics eventually accumulate in the environment [50], causing massive soil and water pollution and posing a direct threat to human survival and health due to the improper handling and low biodegradability of antibiotics [51]. Therefore, how to effectively eliminate antibiotics from environment has become one of the hot spots for researchers. Photocatalytic degradation technology is very beneficial to the degradation of antibiotics in the environment because of its advantages of simplicity of operation, green and low cost [52]. Here the catalytic performance of 3D urchin-like ZnO/Ag HS structures for the degradation of antibiotics has been investigated under visible light irradiation.

**Figure 8:**
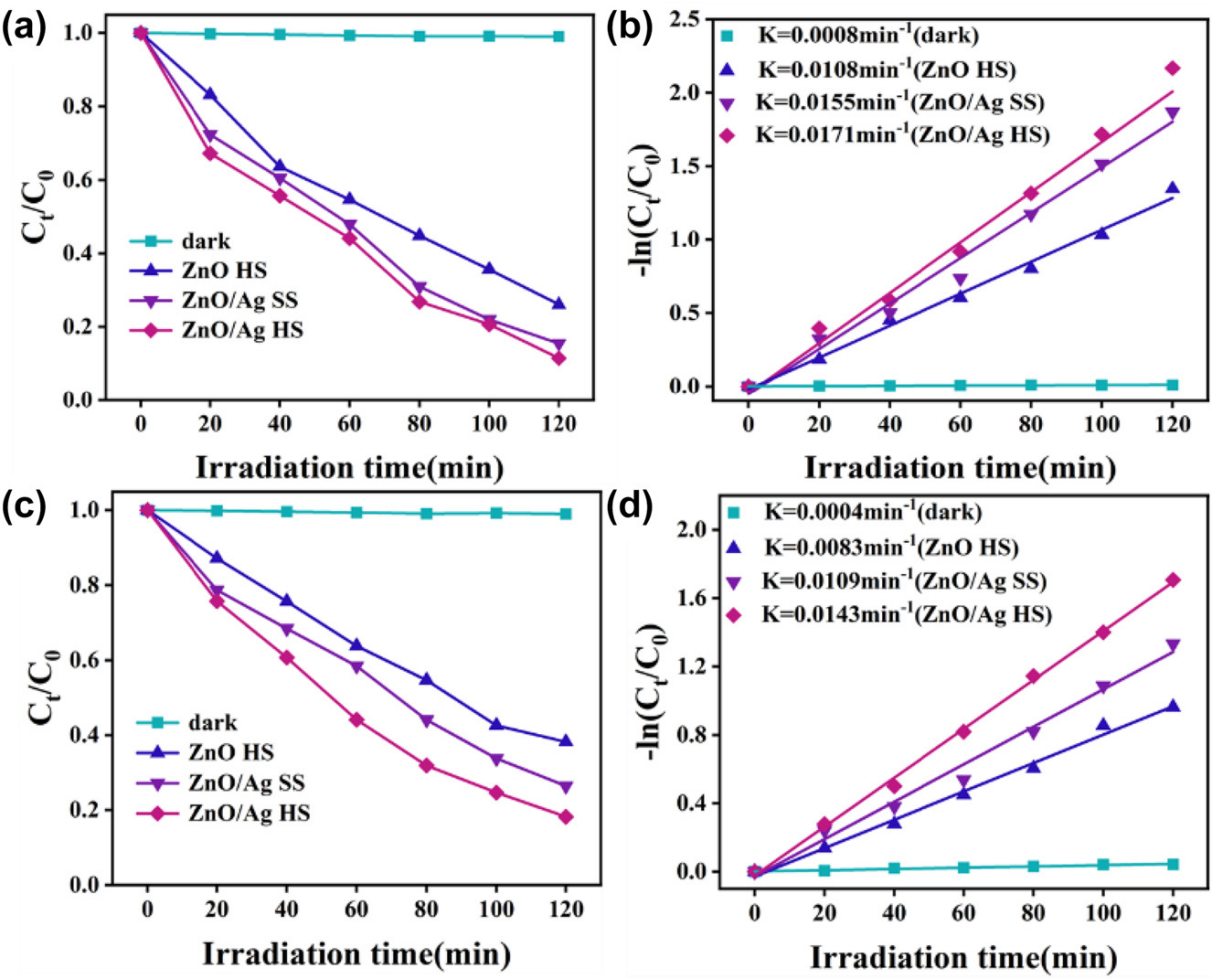
Photocatalytic degradation curves of (a) MNZ and (c) CIP for different samples. The corresponding pseudo-first-order kinetic fitting curves for (b) MNZ and (d) CIP.

As shown in Figure 8a, the concentration of MNZ almost has no difference under black condition. After adding the photocatalyst of ZnO HS, the degradation of MNZ occurred, and the concentration of MNZ solution was gradually reduced with the irradiation time of visible light. And the catalytic efficiency after deposited Ag NPs was better than that of undeposited Ag NPs due to the LSPR effect caused by the collective electron oscillation, which play a key role in the visible light catalytic activity. Furthermore, ZnO/Ag HS exhibited efficient photocatalytic performance than the ZnO/Ag SS profited from the fact that the inner cavity of the HS structure would produce stronger light absorption capacity through reflection and scattering, and the degradation efficiency of ZnO/Ag HS could reach 89 % at 120 min.

To better demonstrate the photocatalytic activity of these photocatalysts, a pseudo-first-order kinetic model was used to study the kinetics of the photocatalytic reaction for the degradation of MNZ with the following equation:

(1) $$-\ln\left(\frac{C_{t}}{C_{0}}\right)=kt$$

(2) $$\left(\frac{1}{C_{t}}\right)-\left(\frac{1}{C_{0}}\right)=kt$$

where *k*, *t*, *C*_*t*_, *C*_0_ represent rate constants, radiation time, concentration corresponding to radiation time, and initial concentration. As shown in Figure 8b, the 3D urchin-like ZnO/Ag HS structure possesses the highest *k* value of 0.0171 min^−1^ compared with ZnO/Ag SS and ZnO HS, further confirm the best photocatalytic activity. In addition, the photocatalyst based ZnO and Ag can also degrade other antibiotics, such as CIP, and the catalytic efficiency was shown in Figure 8c, where the ZnO/Ag HS possesses the most outstanding degradation performance than ZnO/Ag SS and ZnO HS, which the photodegradation efficiency can realize 82 % at 120 min. The efficient photocatalytic performance of the 3D urchin-like ZnO/Ag HS structure can be attributed to its excellent light trapping ability. The pseudo-first-order kinetic model of CIP was shown in Figure 8d, undoubtedly ZnO/Ag HS structure possesses the highest k value indicating the best photodegradation efficiency for CIP. Moreover, the stability of the photocatalyst is also a major indicator for practical applications. We carried out photocatalytic degradation experiment four cycles. As shown in Figure S5, the degradation efficiency of CIP showed no significant decrease (about 5 %), which indicates that the photocatalyst hardly suffer from photo corrosion.

**Figure 9:**
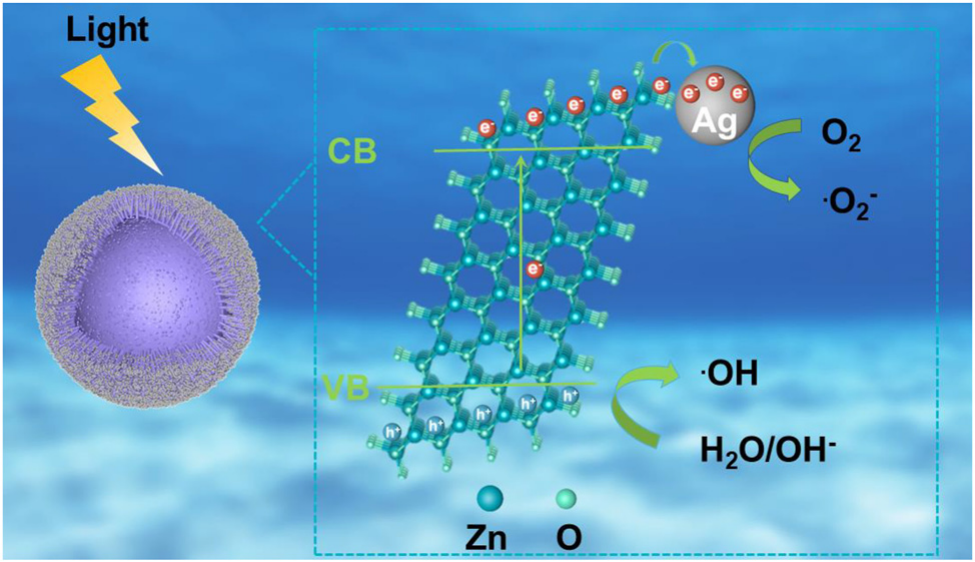
Schematic illustration of the degradation mechanisms of ZnO/Ag HS.

Based on the aforementioned experimental results, the possible mechanism for antibiotics degradation in the presence of 3D urchin-like ZnO/Ag HS structure was illustrated in Figure 9. Under visible light irradiation, the electrons of ZnO becomes stimulated and generates charge carriers, which were produced in response to the light and can move from the valence band (VB) to the conduction band (CB), resulting in vacant space for an electron, also known as “hole.” Therefore, the electrons on the CB of ZnO can produce super-oxide radical anions species. Similarly, the holes on the VB of ZnO can convert H_2_O molecules to hydroxyl radicles. These reactive species i.e. superoxide radical anions and hydroxyl radicles are responsible for the improved degradation of antibiotics to nontoxic products. Due to the difference of work function between Ag and ZnO, the Schottky barrier was formed when Ag was contacted with ZnO. Therefore, electrons will transfer from CB of ZnO to Ag easily, and the formation of Schottky barrier can prevent the recombination of photogenerated charge carriers. Furthermore, the hollow spheres improved the utilization rate of visible light, and extended the lifetime of charge carriers, which greatly improved the photocatalytic activities.

## 4 Conclusions

In summary, combining the urchin-like hollow structure and LSPR effect of Ag NPs, the ZnO/Ag HS possesses excellent SERS and plasma catalytic properties, which is more effective in detection sensitivity and photodegradation efficiency compared with the conventional 2D ZnO/Ag arrays and SS structure. The prepared 3D urchin-like ZnO/Ag HS have super low detection limits, excellent uniformity and reproducibility as well as high catalytic activity under visible light irradiation due to the unique hollow structure, which effectively improves the utilization of light. Experimental and theoretical simulations demonstrate that 3D urchin-like ZnO/Ag HS structure is not only an effective SERS substrate for antibiotic detection, but also a catalyst for antibiotic photodegradation, which can be a viable candidate for ultrasensitive and reproducible environmental remediation.

## Supplementary Material

Supplementary Material Details
