# Supplementary material for: Micro-nano hierarchical urchin-like ZnO/Ag hollow sphere for SERS detection and photodegradation of antibiotics
Source: Nanophotonics. 2024 Jan 23;13(3):307–18. doi: 10.1515/nanoph-2023-0659 (PMC11501311; doi:10.1515/nanoph-2023-0659)
Supplement: Supplementary file 1 — Supplementary Material Details [file j_nanoph-2023-0659_suppl.pdf]

Supplementary Material

Micro-Nano hierarchical urchin-like ZnO/Ag hollow sphere for SERS detection and photodegradation of antibiotics

Yang Jiao[a,&], Yuanyuan Pan[a,&], Moru Yang[a], Zhen Li[a], Jing Yu[a], Rong Fu[b], Baoyuan Man[a], Chao Zhang[a,*] Xiaofei Zhao[a,*]

[a] School of Physics and Electronics, Shandong Normal University, Jinan 250014, China

[b] School of Chemistry and Chemical Engineering, Liaocheng University, Liaocheng 252000, China

[&] These authors contributed equally to the work.

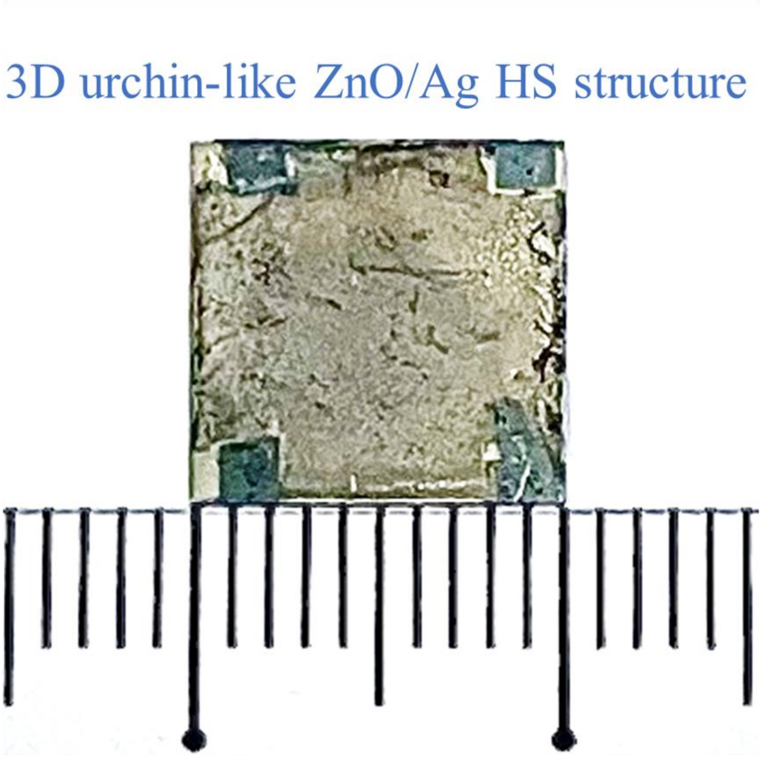

Figure S1 Photograph of fabricated substrates.

* Corresponding author.

E-mail: czsdnu@126.com (Chao Zhang) and zxfsdnu@126.com (Xiaofei Zhao)

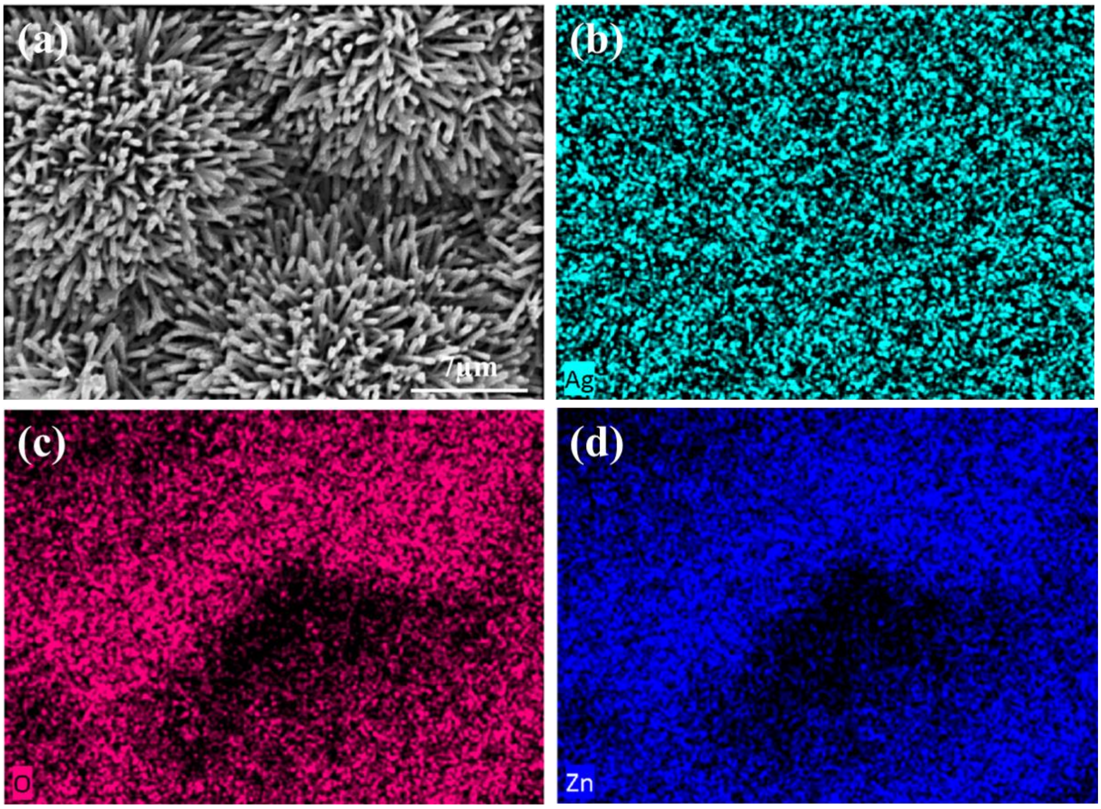

Figure S2 EDS elemental mapping images of an 3D urchin-like ZnO-Ag HS structure from (a) selection region, (b)-(d) illustrate Ag, O and Zn element respectively.

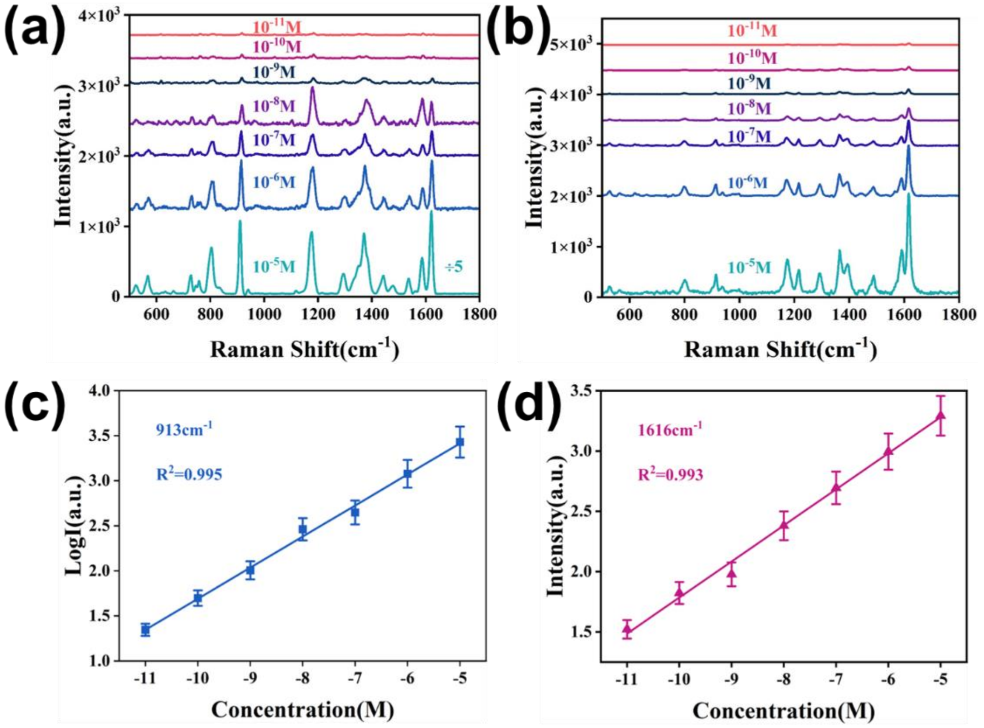

Figure S3 Raman spectra of (a) CV and (b) MG with different concentrations on 3D urchin-like ZnO/Ag HS. (c) The calibration curves of Raman intensity versus CV (10^{-5}-10^{-11}M) at 913 cm^{-1}, (d) Raman intensity versus MG (10^{-5}-10^{-11}M) at 1616 cm^{-1}.

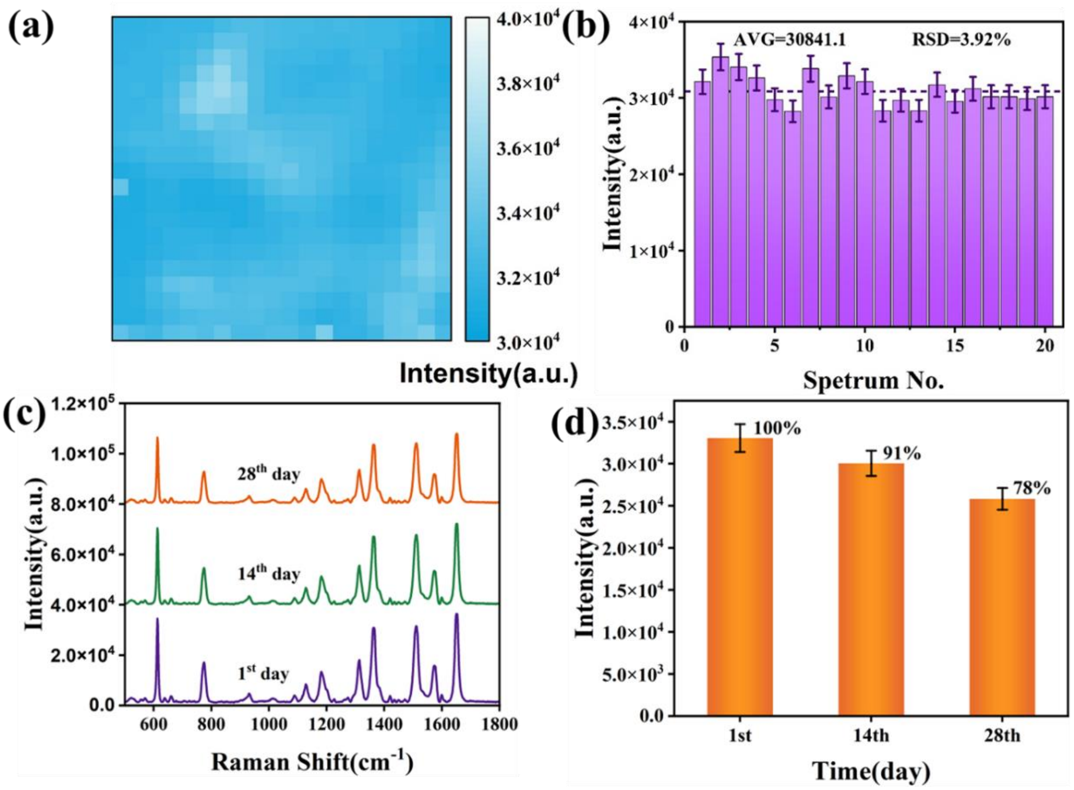

Figure S4 (a) Raman intensity mapping of 10^{-5} M R6G at 613 cm^{-1} on 3D urchin-like ZnO/Ag HS substrates with a step size of 2 μm. (b) Intensity distribution of R6G at 613 cm^{-1} peaks detected from 20 batches substrate. (c) Raman intensity of 10^{-5} M R6G on 3D urchin-like ZnO/Ag HS substrates. Lasts 28 days, with 14-day intervals. (d) The corresponding Raman intensity of at 613 cm^{-1}.

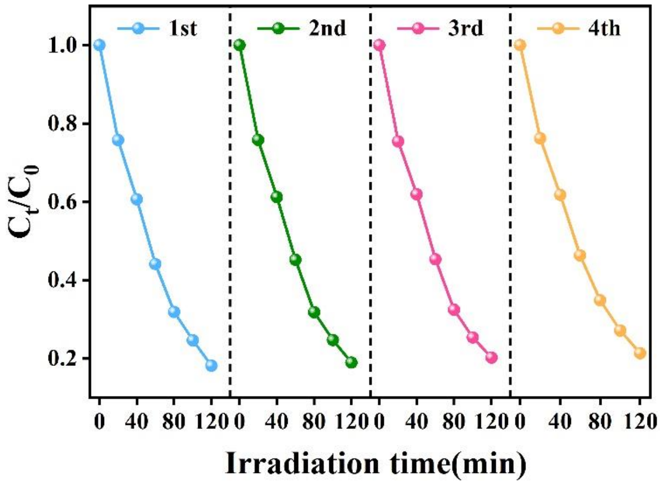

Figure S5 CIP photocatalytic degradation cycle experiment.
